# Supplementary material for: Wheat ABA Receptor TaPYL5 Constitutes a Signaling Module with Its Downstream Partners TaPP2C53/TaSnRK2.1/TaABI1 to Modulate Plant Drought Response
Source: Int J Mol Sci. 2023 Apr 27;24(9):7969. doi: 10.3390/ijms24097969 (PMC10178726; doi:10.3390/ijms24097969)
Supplement: Supplementary file 1 [file ijms-24-07969-s001.zip › ijms-2348020-supplementary.pdf]

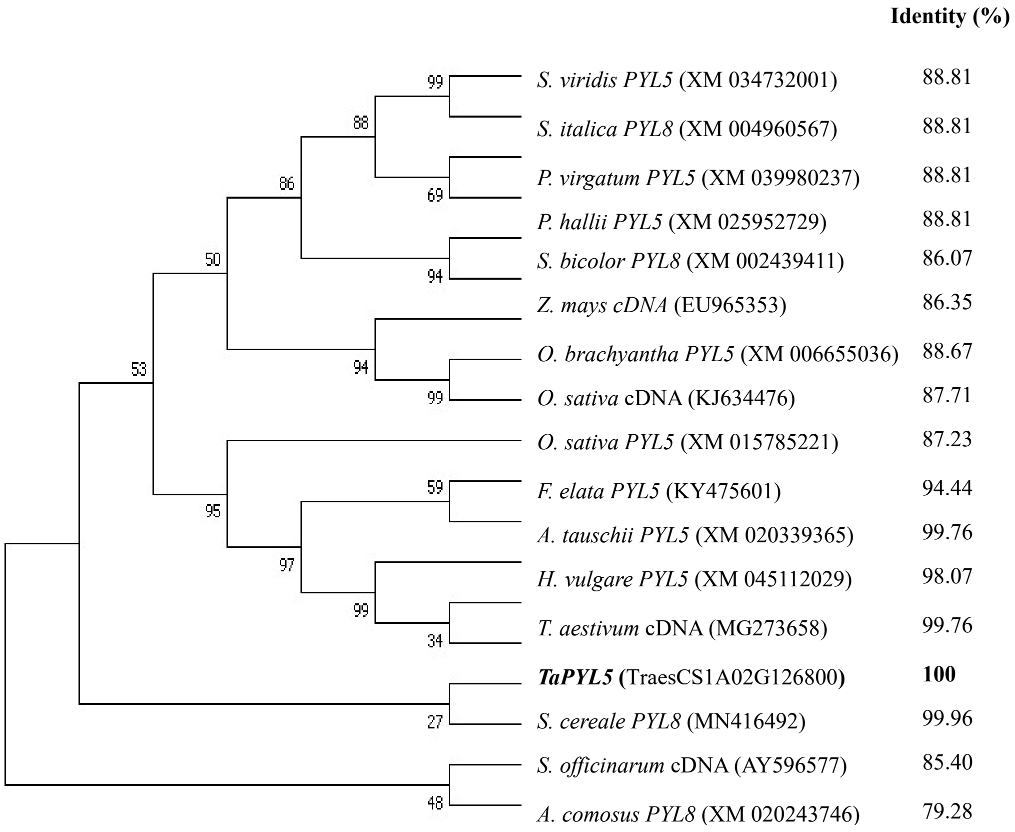

Supplementary Figure S1. Phylogenetic relations among *TaPYL5* and its homologous genes distributed in various plant species.

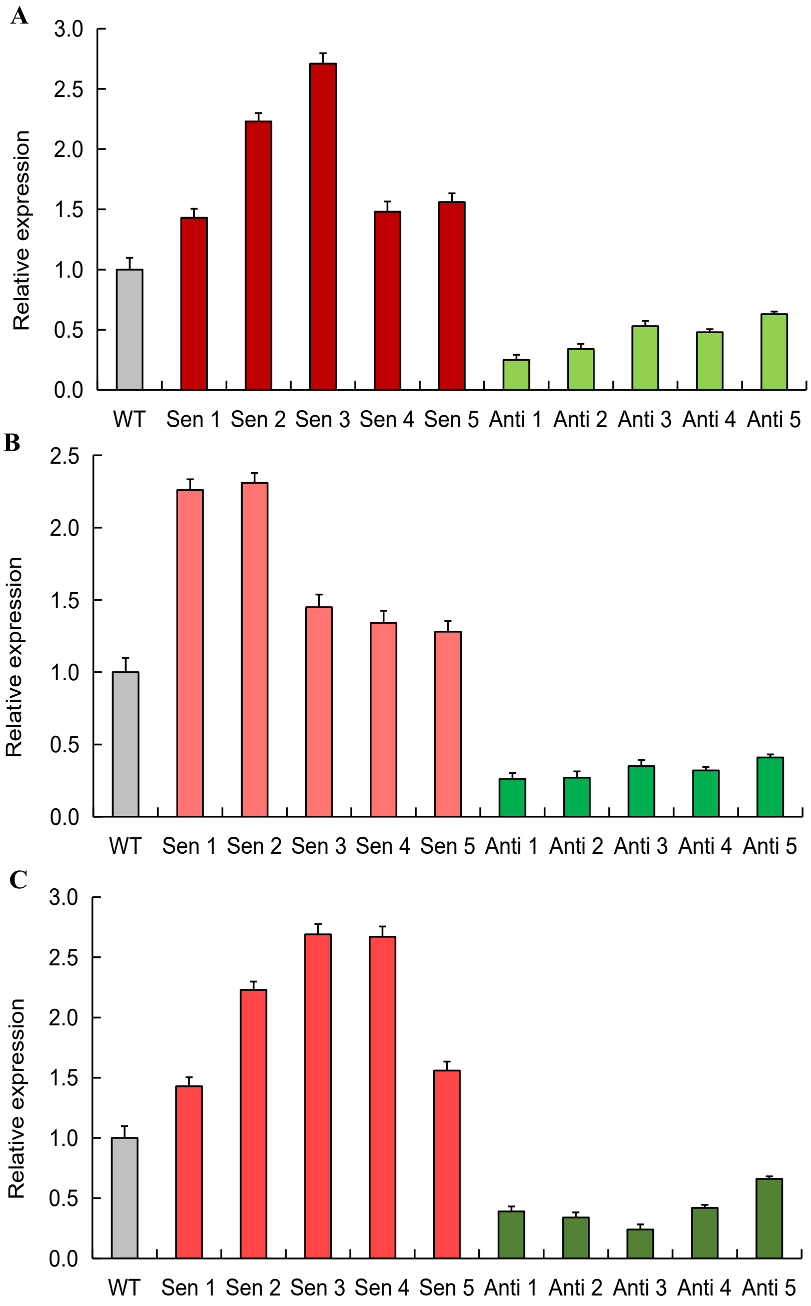

Supplementary Figure S2. Expression levels of the target gene in *TaPYL5, TaPPP2C53* and *TaSnRK2.1* transgenic wheat lines. (**A**) Expression levels of *TaPYL5* in transgenic lines. (**B**) Expression levels of *TaPP2C53* in transgenic lines. (**C**) Expression levels of *TaSnRK2.1* in transgenic lines. WT, wild type. Data are shown by average from triplicate results plus standard error. The expression values were normalized by *Tatubulin*, a constitutive gene in *T. aestivum* species whose expression level was set as 1.

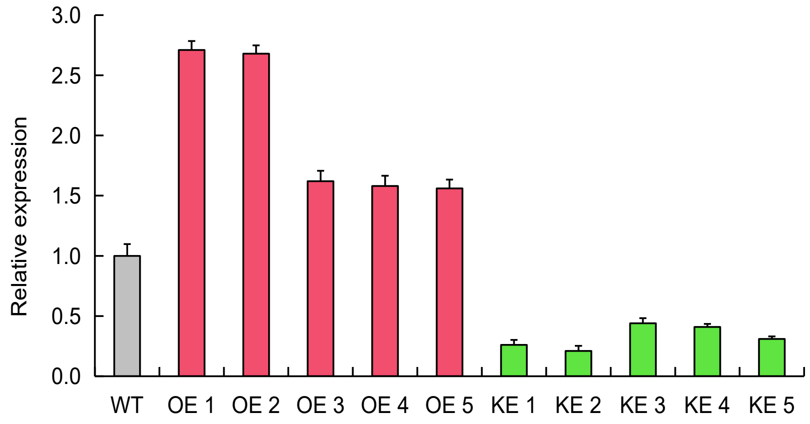

Supplementary Figure S3. Expression levels of the target gene in *TaABI1* transgenic wheat lines. Data are shown by average from triplicate results plus standard error. The expression values of target gene were normalized by *Tatubulin*, a constitutive gene in *T. aestivum* species whose expression level was set as 1.

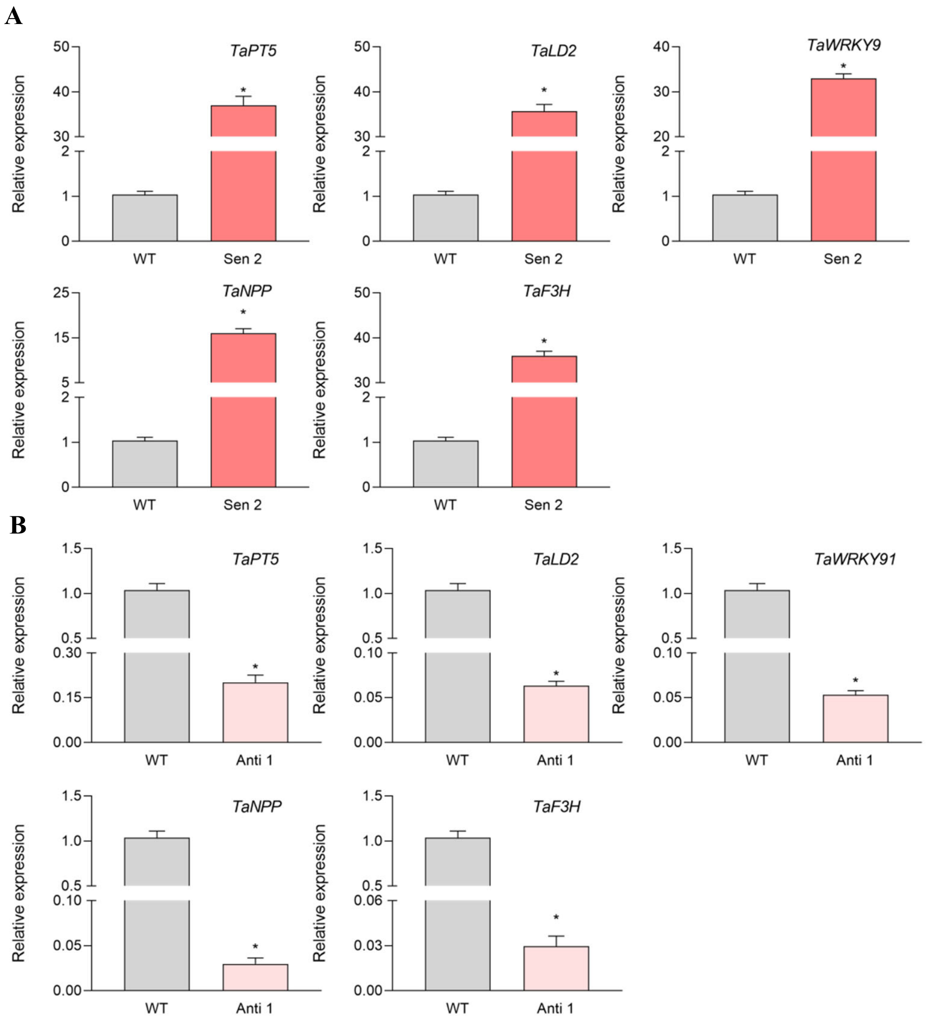

Supplemental Figure S4. qRT-PCR results in roots of *TaPYL5* transgenic wheat lines for the differentially expressed genes with upregulated expression pattern identified based on RNA-seq analysis. (**A**) Expression levels in Sen 2. (**B**) Expression levels in Anti 1. In (**A**,**B**): *TaPT5,* potassium transporter 5-like gene (TraesCS3D02G439300), *TaLD2*, leucoanthocyanidin dioxygenase 2-like gene (TraesCS6D02G004300), *TaWRKY9*, transcription factor WRKY9 gene (TraesCS6A02G326500), *TaNPP*, purple acid phosphatase gene (TraesCS4B02G348600), *TaF3H*, flavanone 3-hydroxylase gene (TraesCS2A02G493500). Sen 2, a transgenic line overexpressing *TaPYL5*. Anti 1, a transgenic line with *TaPYL5* knockdown expression. The constitutive gene *Tatubulin* was used as internal standard to normalize target gene transcripts, whose expression level was set as 1. *: significant difference from the WT (P<0.05).

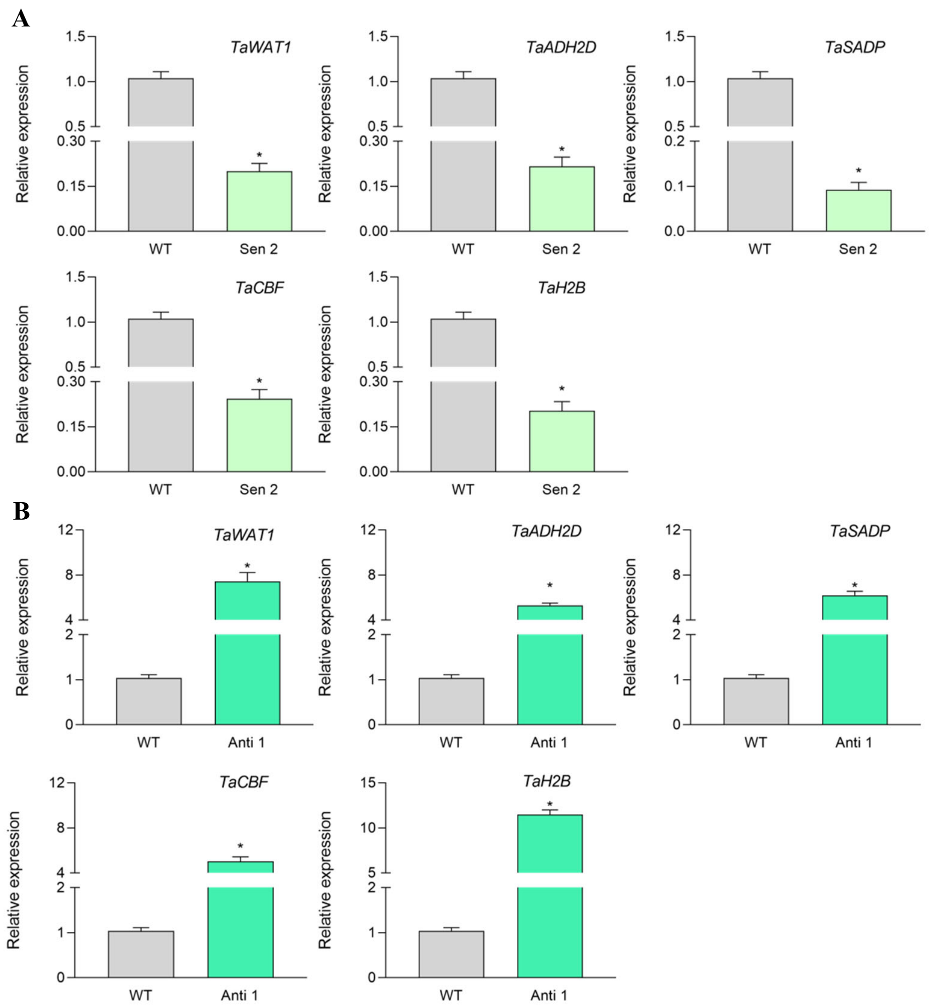

Supplemental Figure S5. qRT-PCR results in roots of *TaPYL5* transgenic wheat lines for the differentially expressed genes with downregulated expression pattern identified based on RNA-seq analysis. (**A**) Expression levels in Sen 2. (**B**) Expression levels in Anti 1. In (**A**,**B**): *TaWAT1,* WAT1-related protein gene (TraesCS3B02G006900), *TaADH2D*, alcohol dehydrogenase ADH2D gene (TraesCS4A02G202300), *TaSADP*, S-adenosylmethionine decarboxylase proenzyme gene (TraesCS6D02G202500), *TaCBF*, AP2 domain CBF protein gene (TraesCS6D02G238200), *TaH2B*, histone H2B gene (TraesCS5D02G063200). Sen 2, a transgenic line overexpressing *TaPYL5*. Anti 1, a transgenic line with *TaPYL5* knockdown expression. The constitutive gene *Tatubulin* was used as internal standard to normalize target gene transcripts, whose expression level was set as 1. *: significant difference from the WT ($P<0.05$).

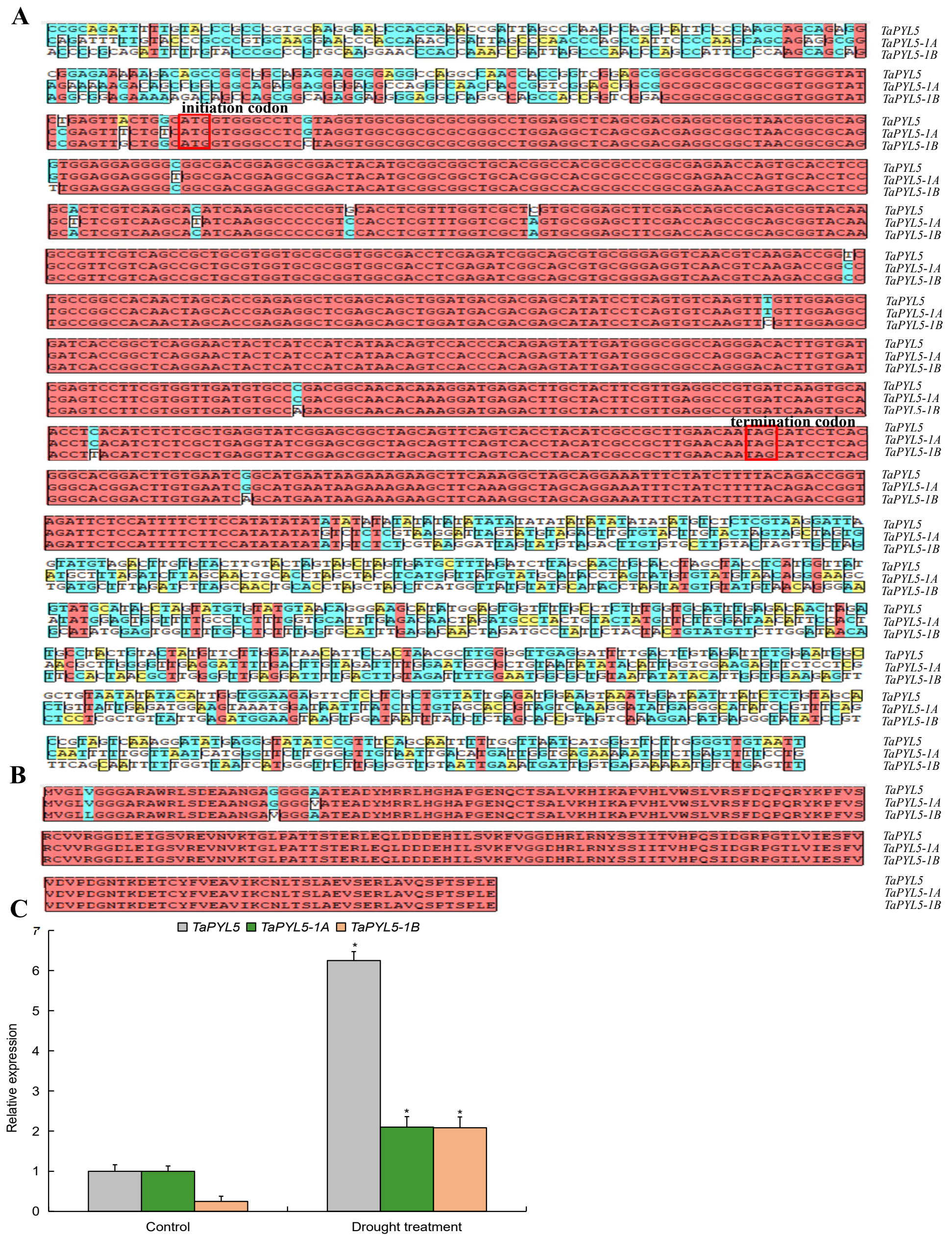

Supplemental Figure S6. Characterization on *TaPYL5* and its homeologs. (**A**) Alignment results among *TaPYL5* and its homeologs at nucleic acid level. (**B**) Alignment results among TaPYL5 and its homeologous proteins situated on 1A and 1B. (**C**) Expression levels of *TaPYL5* and its homologs in roots under normal growth and drought treatment. In (**A**,**B**), the sequence alignment analyses were conducted based on MEGA8. In (**C**), root tissues of wheat seedlings (cv. Shimai 26) at the third-leaf stage grown in standard MS solution (control) and modified MS solution provided by PEG-6000 (10%, w/v) were collected and subjected to qRT-PCR analysis using specific primers. Constitutive gene *Tatubulin* was used as an internal standard to normalize the target transcripts, whose expression level was set as 1. *: significant difference compared with Control ($P<0.05$).

Supplementary Table S1. PCR primers used in this study

Purpose	Accession number	Forward primer (5´-)	Reverse primer (5´-)
TaPYL5 expression	TraesCS1A02G126800	ATGGTGGGCCTCGTAGGTGGCGG	CTATTGTTCAAGCGGCGATGTAGGT
Tatubulin expression	U76558	CATGCTATCCCTCGTCTCGACCT	CGCACTTCATGATGGAGTTGTAT
TaPYL5 location	XM_037633867	AAAGGTACCATGGTGGGCCTCGTAGGTGGCGG	AAAAAGCTTCTATTGTTCAAGCGGCGATGTAGGT
TaPYL5 overexpression	XM_037633867	TTTCCATGGTGGGCCTCGTAGGTG	TTTGGTAACCTATTGTTCAAGCGGCGA
TaPYL5 knockdown expression	XM_037633867	AAACCATGGCAAAGAGTATCACAGCA	AAAGGTAACCAGTGCACCTCCGCA
TaPYL5 bait in two-hybrid	XM_037633867	AAAGAATTCATGGTGGGCCTCGTAGGTGGCGG	AAAGGATTCCTATTGTTCAAGCGGCGATGTAGGT
TaPP2C6 prey in two-hybrid	TraesCS1A02G358600	TTTGAATTCATGGCGGCGGCGGCGGCG	TTTCTGCAGGTCAGCTTTGCTCATCCG
TaPP2C8 prey in two-Hybrid	TraesCS1A02G441200	TTTGAATTCATGGACGCCCTAGGCGCC	TTTCTGCAGGCTATCTGTTGTTGTTGT
TaPP2C9 prey in two-hybrid	TraesCS1B02G375100	TTTGAATTCATGGCGGCGGCGGCGGCG	TTTCTGCAGGTTAGGAATTATTCTTGG
TaPP2C50 prey in two-Hybrid	TraesCS1D02G363500	TTTGAATTCATGGCGGCGGCGGCGGCC	TTTCTGCAGGTTAGGAATTATTCTTGG
TaPP2C53 prey in two-hybrid	TraesCS1A02G441200	TTTGAATTCATGGACGCCCTAGGCGCC	TTTCTGCAGGCTATCTGTTGTTATTGT
TaSnRK2.1 prey in two-hybrid	TraesCS2A02G493800	TTTGAATTCCATGGATCGGTACGAGGTG	TTTGGATCCTCACAACGGGCACACGAAA
TaSnRK2.2 prey in two-hybrid	TraesCS2A02G163800	TTTGAATTCATGGAGCGGTACGAGGTG	TTTGGATCCTCACAACGCGCACACGAA
TaSnRK2.3 prey in two-hybrid	TraesCS1A02G215900	TTTGAATTCATGGAGGAGAGGTACGAG	TTTGGATCCTCAGTAGGTCTCCCCCTC
TaSnRK2.4 prey in two-hybrid	TraesCS3A02G381100	TTTGAATTCATGGAGAAGTACGAGGCG	TTTGGATCCTCATGATATGCGTAGCGA
TaSnRK2.5 prey in two-hybrid	TraesCS2A02G566700	TTTGAATTCATGGAGAAGTACGAGCCGG	TTTGGATCCTCAGATTTGGAGCTTGCT
TaSnRK2.6 prey in two-hybrid	TraesCS2A02G303900	TTTGAATTCATGGAGAGGTACGAGCTG	TTTGGATCCCTAGCTGATGTGGAACTC
TaSnRK2.7 prey in two-hybrid	TraesCS1A02G270800	TTTGAATTCATGGACAAGTACGAGGAGG	TTTGGATCCTTAGATGTGCAACACGCT
TaPP2C53 expression	TraesCS1A02G441200	ATGGACGCCCTAGGCGCC	CTATCTGTTGTTGTTGTCAG
TaPP2C53 overexpression	TraesCS1A02G441200	AAACCATGGATGGACGCCCTAGGCGCC	AAAGGTAACCCTATCTGTTGTTGTTGT
TaPP2C53 knockdown expression	TraesCS1A02G441200	AAACCATGGATGGACGCCCTAGGCGCC	AAAGGTAACCCTAGGCGCCGCGCTGCC
TaSnRK2.1 expression	TraesCS2A02G493800	GGGACATAGGAAGAAAGAGGG	GGAAGCATGACAAAACAGGGAT
TaSnRK2.1 overexpression	TraesCS2A02G493800	AAAAGATCTATGGATCGGTACGAGGTG	AAAGGTAACCACTCATGAGCAAT
TaSnRK2.1 knockdown expression	TraesCS2A02G493800	AAAAGATCTATGACAAAACAGGGATCC	AAAGGTAACCAAGGAGCACTT
TaABI1 expression	AB238930	ATGGAGGACGTGGCCGTG	GGAAGTTCAAGAGCAAAACTTAA

TaABI1 overexpression	AB238930	AAACCATGGATGGAGGACGTGGCCGTG	AAAGGTAACCTTAAGTTTTGCTCTTGAACTTCC
TaABI1 knockdown expression	AB238930	AAACCATGGATGTTGTCCTTGCTCCCC	AAAGGTAACCTTAGCCCCGCCACGGCC
TaPYL5-BiFC	XM_037633867	AAAGGATCCATGGTGGGCCTCGTAGG	AAAGGTACCGTATTGTTCAAGCGGCG
TaPP2C53- BiFC	TraesCS1A02G441200	AAAGGATCCATGGACGCCCTAGGCGC	AAAGTCGACGTATCTGTTGTTATTGT
TaSnRK2.1- BiFC	TraesCS2A02G493800	AAAGGATCCATGGATCGGTACGAGGTG	AAAGTCGACCACTCATGAGCAAT
*TaPYL5*Pro-D1	XM_037633867	TTGGATCCGGTCATGTGTCACATGACC	TTGTCGACGCCAGTAACTCAGATACC
*TaPYL5*Pro-D2	XM_037633867	TTGGATCCGGGCGAAGGGATAGAAAC	TTGTCGACGCCAGTAACTCAGATACC
*TaPYL5*Pro-D3	XM_037633867	TTGGATCCGAAGATGGTGCATGGTTGG	TTGTCGACGCCAGTAACTCAGATACC
*TaPYL5*Pro-D4	XM_037633867	TTGGATCCGCCCGCGGTAGTTTTCCC	TTGTCGACGCCAGTAACTCAGATACC
*TaPYL5*Pro-D5	XM_037633867	TTGGATCCCCTCCGCGACGAGATGGTCC	TTGTCGACGCCAGTAACTCAGATACC
TaPT5 expression	TraesCS3D02G439300	ATGGCGGTGCCTCTGAAAGC	CTAGATCTCATATGTCATACCC
TaLD2 expression	TraesCS6D02G004300	ATGGCGCGCGTGGAGGCGC	TTAATTAACTTCCACTAGGTTGGCT
TaWRKY9 expression	TraesCS6A02G326500	ATGTCATCATCAAAGAGG	TCAACCACCCCCCCACTTGT
TaNPP expression	TraesCS4B02G348600	ATGGGGATGGCGCATGTCGC	CCACCACACTCGCTAGCTGA
TaF3H expression	TraesCS2A02G493500	ATGGCGCCGGTGAGCAACGAG	TCAGGCAAGAATTTCGTTGAGAGA
TaWAT1 expression	TraesCS3B02G006900	ATGGCGGACGCGGTGGATG	TCAGACATTGTCGGAGGTGGT
TaADH2D expression	TraesCS4A02G202300	ATGGCGGACGCGGTGGATG	TCAGACATTGTCGGAGGTGGT
TaSADP expression	TraesCS6D02G202500	ATGGCCGTCTCTGCGATTGG	TAAGAAGATGAAGAGCTCCTGA
TaCBF expression	TraesCS6D02G238200	ATGGACCTCAGTGCCCTC	TCAGTAGCTCCACAAGCTGACC
TaH2B expression	TraesCS5D02G063200	ATGGCCCCCAAGGCGGAGAAG	CAAGTTCACCTCCTCCTAG

Supplementary Table S2. Drought-related *cis*-acting elements in the *TaPYL5* promoter

<table>
<tr><th>Site Name</th><th>Organism</th><th>Position</th><th>Matrix score.</th><th>Species</th><th>Function</th></tr>
<tr><td>MBS</td><td>CAACTG</td><td>1394</td><td>-</td><td rowspan="2">Arabidopsis thaliana</td><td rowspan="2">MYB binding site involved in drought-inducibility</td></tr>
<tr><td>MYB recognition site</td><td>CCGTTG</td><td>577</td><td>-</td></tr>
<tr><td>MYB-binding site</td><td>CAACAG</td><td>997</td><td>-</td><td>Nicotiana tabacum</td><td rowspan="9">cis-acting regulatory element</td></tr>
<tr><td>MYB</td><td>CAACTG</td><td>1394</td><td>-</td><td rowspan="8">Arabidopsis thaliana</td></tr>
<tr><td rowspan="4">MYB</td><td>CAACCA</td><td>974</td><td>-</td></tr>
<tr><td>CAACAG</td><td>997</td><td>-</td></tr>
<tr><td rowspan="2">CAACCA</td><td>1367</td><td>-</td></tr>
<tr><td>1949</td><td>+</td></tr>
<tr><td rowspan="3">DRE core</td><td rowspan="3">GCCGAC</td><td>257</td><td>-</td></tr>
<tr><td>1415</td><td>-</td></tr>
<tr><td>1680</td><td>+</td></tr>
<tr><td rowspan="2">ABRE</td><td rowspan="2">ACGTG</td><td>437</td><td>+</td><td rowspan="2">Arabidopsis thaliana</td><td rowspan="2">cis-acting element involved in the abscisic acid responsiveness</td></tr>
<tr><td>1293</td><td>-</td></tr>
</table>
